# Amino Acid Synthesis in a Supercritical Carbon Dioxide - Water System

**DOI:** 10.3390/ijms10062722

**Published:** 2009-06-15

**Authors:** Kouki Fujioka, Yasuhiro Futamura, Tomoo Shiohara, Akiyoshi Hoshino, Fumihide Kanaya, Yoshinobu Manome, Kenji Yamamoto

**Affiliations:** 1 International Clinical Research Centre, Research Institute, International Medical Centre of Japan, 1-21-1 Toyama, Shinjuku-ku, Tokyo, Japan; E-Mails: kfujioka@ri.imcj.go.jp (K.F.); hoshinoa@nih.go.jp (A.H.); 2 Department of Molecular Cell Biology, Institute of DNA Medicine, Jikei University School of Medicine, 3-25-8 Nishi-shimbashi, Minato-ku, Tokyo, Japan; E-Mail: manome@jikei.ac.jp; 3 Sekisui Chemical Co., Ltd., 2-1 Hyakuyama, Shimamoto-cho, Mishima-gun, Osaka, Japan; E-Mail: shiohara002@sekisui.jp

**Keywords:** supercritical carbon oxide, amino acid, prebiotic chemistry, soda fountain, Mars

## Abstract

Mars is a CO_2_-abundant planet, whereas early Earth is thought to be also CO_2_-abundant. In addition, water was also discovered on Mars in 2008. From the facts and theory, we assumed that soda fountains were present on both planets, and this affected amino acid synthesis. Here, using a supercritical CO_2_/liquid H_2_O (10:1) system which mimicked crust soda fountains, we demonstrate production of amino acids from hydroxylamine (nitrogen source) and keto acids (oxylic acid sources). In this research, several amino acids were detected with an amino acid analyzer. Moreover, alanine polymers were detected with LC-MS. Our research lights up a new pathway in the study of life’s origin.

## 1. Introduction

Prebiotic amino acids syntheses have attracted the attention of scientists [1], since amino acids are one of the essential materials for chemical evolution in early Earth. The prebiotic synthesis by electric discharge in reducing gasses (CH_4_, NH_3_, H_2_, and H_2_O) through Miller’s experiment has given experimental support to the chemical evolution [2]. After Miller’s report, many prebiotic syntheses in various types of geological environments have been examined, including volcanoes [3], hydrothermal system [4–8], meteorites[9,10], and other planetary bodies [11].

In the recent research, however, the early Earth atmosphere is thought to have had a moderately oxidative state, being mostly composed of CO_2_ and N_2_ [12]. There are few reports on the synthesis of organic molecules including amino acids under such a condition [13]. For this reason, the fundamental question about the origin of life – how organic molecules appeared in the early Earth – nowadays is still open. From the early Earth atmosphere, we focused on CO_2_, which is also a component of the atmosphere of Mars, as a reaction medium. CO_2_ is known to change directly from solid phase to gas phase through sublimation at −78.51 ºC and normal pressure on Earth. Liquid carbon forms only above the triple point (−56.6 ºC, 0.52 MPa). The critical point of CO_2_ is known at 31.1 ºC, 7.38 MPa and supercritical CO_2_ is reactive towards other molecules.

Considering the reactivity, we assumed that the supercritical CO_2_ in soda fountains in the crusts on early Earth and Mars affected amino acid synthesis. For the existence of such soda fountains, there are two essential factors: (1) abundant CO_2_ (carbonate) and H_2_O and (2) late stage of volcanoes. Although McKay *et al.* reported the detection of polycyclic aromatic hydrocarbons in the Martian meteorite ALH84001 as a clue of organic synthesis [14], is there any evidence of soda fountains on Mars? Different from the Earth, on Mars the main component of the atmosphere is CO_2_. Moreover, carbonate globules were also detected in the Martian meteorite. Additionally, like on Earth, H_2_O was present abundantly on early Mars, since the vestiges of rivers were found on the over-3-Gyr ground [15]. On the other hand, there are thought to have been some active volcanoes 3.5–2.5 Gyr before, due to the volcanism simulation based on the model of Martian mantle convection [16]. Therefore soda fountains could existence on early Earth or Mars. To the best of our knowledge, no previous study has examined soda fountains in the crusts as a reaction medium for producing organic molecules necessary for the origin of life.

The principal objective of this study was to determine the possibility of amino acid synthesis in the co-existence of supercritical CO_2_ and liquid H_2_O, mimicking crust soda fountains which represent more moderate conditions than supercritical H_2_O. Our research does not contradict other hypotheses of the life’s origin and we assumed that simple organic molecules from other prebiotic syntheses enter to our synthesis system. Therefore we chose hydroxylamine and pyruvic acid as the primary reactants, which could be synthesized in other prebiotic synthesis [17,18]. In this paper, we demonstrated that the co-existence of supercritical CO_2_ and liquid H_2_O could produce some amino acids and polymers from the simple organic molecules.

## 2. Results and Discussion

### 2.1. Amino acid synthesis in supercritical carbon dioxide

**Figure 1.**
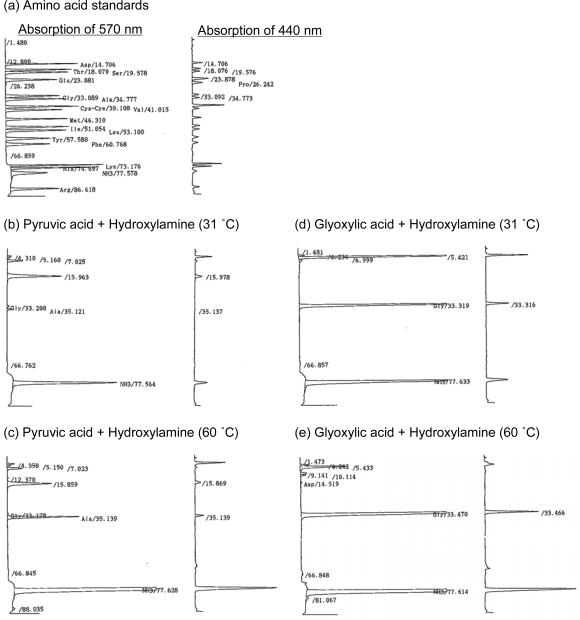
Chromatograms of amino-acid analysis. (a) Amino acid standards. (b) Pyruvic acid + Hydroxylamine at 31 ºC. (c) Pyruvic acid + Hydroxylamine at 60 ºC. (d) Glyoxylic acid + Hydroxylamine at 31 ºC. (e) Glyoxylic acid + Hydroxylamine at 60 ºC.

We investigated the possibility that amino acids can be synthesized from hydroxylamine and pyruvic acid or glyoxylic acid, under supercritical CO_2_ conditions (60 ºC). Additionally, in order to compare the conditions, we repeated the same reaction under subcritical CO_2_ conditions (31 ºC) as a control. For the detection of amino acids, an amino acid analyzer was used (Figure 1). Since this analysis system is adjusted for detecting amino-acid monomers, the differences before and after the hydrolysis reaction indicates the amount of polymer hidden in the original products.

Using pyruvic acid as carbon sourcs, two kinds of amino acids, alanine and glycine, were detected at 60 ºC and 31 ºC (Table 1). The free alanine yield at 60 ºC (60.7 μmol) was higher than that at 31 ºC (4.1 μmol), whereas the free glycine yield at 60 ºC (0.99 μmol) was lower than that at 31 ºC (2.2 μmol). These results suggested that alanine synthesis was conducted more effectively at 60 ºC.

After hydrolysis treatment with 6N HCl, 256.2 μmol and 70.2 μmol of alanine were detected at 60 ºC and 31 ºC, respectively (Table 1). Additionally, arginine (0.32 μmol) was detected at 60 ºC. From the significant differences of concentrations between before and after hydrolysis reaction, alanine polymers were synthesized in larger quantity at 60 ºC. However, the ratios of free alanine: alanine polymers, were 1:3.2 at 60 ºC; 1:16.5 at 31 ºC. Considering these results, alanine polymers may be more stable in lower temperature or degraded at high temperature.

In order to estimate the mechanism of alanine synthesis, we compared the reactions using glyoxylic acids as another carboxylic acid source. Different from the reaction using pyruvic acid, free alanine was not detected at both temperatures, whereas free glycine was detected at 60 ºC (866.3 μmol) and 31 ºC (183.3 μmol); free aspartic acid (2.5 μmol) was detected at 60 ºC (Table 1). These results suggested that the methyl group of alanine synthesized from pyruvic acid were derived from the methyl group of pyruvic acid, and that the alanine was synthesized from hydrogenation of pyruvic acid oxime, since alanine was not detected in the glyoxylic acid reaction.

Some methods have been proposed for the synthesis of alanine from pyruvic acid oxime. Hamlin *et al*. reported a hydrogenation of the oxime with a palladium-charcoal catalyst [19], and Borszeky *et al*. reported that a hydrogenation of pyruvic acid oxime with palladium/alumina catalysts provided the high yields of racemic alanine [20]. Although these hydrogenation were conducted with catalysts, we speculated that oxime hydrogenation also occurred under our supercritical CO_2_ conditions.

After the hydrolysis of the product from glyoxylic acid, glycine (819.2 μmol) and aspartic acid (1.8 μmol) were detected at 60 ºC, whereas glycine (209.2 μmol) was detected at 31 ºC reaction (Table 1). Additionally, 1/2-cystine (1.2 μmol) and leucine (0.37 μmol) was detected at 60 ºC and 31 ºC, respectively (data not shown). The sulfur atoms of leucine and 1/2-cystine may be derived from impurities of the product of hydroxylamine hydrochloride (see Experimental Section).

The main detected amino acids both before/after hydrolysis conditions, alanine, glycine, and aspartic acid, are GNC code encoding amino acids (N means either of four bases). Ikehara *et al*. proposed in their hypothesis a [glycine, alanine, aspartic acid, and valine (GADV)]-protein world hypothesis on the origin of life, based on the GNC code encoding amino acids as the most primitive genetic code [21]. These GADV amino acids are known to be detected in the Miller discharge experiment [2] and the Miller volcanic spark discharge experiment using his apparatus performed after after his death [22]. Although valine was not detected in our experiments using pyruvic acid or glyoxylic acid, as a hypothesis, if the carboxylic acid source is changed to α-ketoisovaleric acid (3-methyl-2-oxobutanoic acid), valine may be synthesized under supercritical CO_2_ conditions. Our data in CO_2_ were thus partially agreement with the GADV-protein world hypothesis.

Surprisingly, in the glyoxylic acid reaction, the concentrations of glycine before and after hydrolysis reaction did not change dramatically (after hydrolysis reaction: before hydrolysis reaction = 209.2 μmol:183.3 μmol at 31 ºC; 819.2:866.3 at 60 ºC). Comparing the alanine data in the pyruvic acid reaction, it was estimated that glycine polymers were more unstable under supercritical or subcritical CO_2_ conditions, since the ratio of alanine polymers was higher than that of free alanine in the above experiments. In contrast with the results, our previous study showed that glycine polymers, including decaglycine, were obtained from glycine monomer under hydrothermal condition (270 ºC, 10 MPa, and 27-second reaction) with an adiabatic expansion cooling system [23]. The cooling system may be an essential condition to obtain unstable glycine polymers. We speculated that alanine polymers were stable due to the formation of helix forms [24,25], but glycine polymers were unstable due to the large flexibility of the residues under CO_2_ conditions, like in the aqueous model [25].

### 2.2. Analysis of alanine polymer

**Figure 2.**
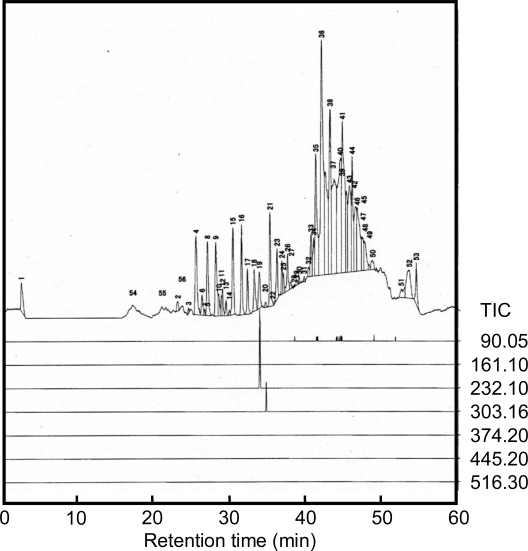
Total ion chromatogram (TIC) and MS chromatograms of alanine monomer and polymers.

In order to confirm alanine polymer synthesis under supercritical CO_2_ conditions, we analyzed the sample from pyruvic acid at 60 ºC with a LC-MS. We obtained 56 peaks under the gradient LC conditions used (Figure 2). Among these peaks, the positive ions, [M+H]^+^, of 232.13 (alanine trimer) and 303.16 (alanine tetramer) were detected at 33.7 and 34.6 min retention time, respectively (Table 2). The alanine dimer and 5 – 7 mers were not detected. These results suggested that alanine trimer and tetramer were stable under CO_2_ supercritical conditions. From the ESR spectroscopic data, several alanine-based forms can adopt the 3_10_ helix formation in aqueous solution [26,27]. Also under these supercritical conditions, the tetramers could be stable by forming the 3_10_ helix and were thus detected in the aqueous sample due to their stability. However, we could not found out the reason why the trimer was stable under CO_2_ supercritical conditions. We speculate that the trimer was a metastable structure in the supercritical CO_2_; therefore the trimer was synthesized directly from three alanine monomers, not through the dimer structure, and the tetramer was synthesized from a trimer + monomer reaction. The polymers higher then pentamer may be separated out as deposits from supercritical CO_2_. This deposition polymerization in supercritical CO_2_ is known in ethylene polymerizations [28,29]. At the present, it is outside the scope of our research to investigate the deposition, but it will be the subject in future studies.

## 3. Experimental Section

### 3.1. CO_2_ reaction conditions

**Figure 3.**
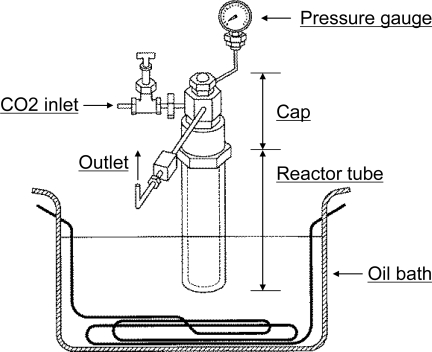
Diagram of the autoclave reactor for the supercritical CO_2_ reaction.

The reactor was made of stainless steel and was connected to a CO_2_ inlet and outlet that have valves and a pressure gauge (Figure 3). Twenty six mmol of hydroxylamine hydrochloride (>97%; Tokyo Chemical Industry Co. Ltd.) and 26 mmol of pyruvic acid (98%; Alfa Aesar) or glyoxylic acid monohydrate (98%; Alfa Aesar) were mixed with 3 mL of Milli-Q (Millipore) water gently in a glass tube. The hydroxylamine hydrochloride could contain the following impurities: sulfate: max. 0.005%; copper: max. 2 ppm; lead: max. 2 ppm; iron: max. 5 ppm. After the glass tube was inserted into the stainless tube, the stainless tube was capped and CO_2_ liquid was injected to purge the air inside. Then 20 g of CO_2_ liquid was injected through the inlet of the reactor contained in an ice box. In order to compare the reaction conditions, we conducted reactions under two sets of conditions: supercritical condition (60 °C, over 7.4 MPa) and subcritical condition (31 °C, less than 7.2 MPa). This subcritical condition was chosen on the basis of our hypothesis that subcritical CO_2_ conditions near the supercritical conditions are most effective for the reactions. The reactor was placed in an oil bath for two h in each condition. After the reactions, the reactors were rapidly moved into an ice box and the CO_2_ gas released. One mL of Milli-Q water was added into the glass tube, and all the samples were transferred to a new glass bottle.

### 3.2. Amino-acid analysis

The aqueous samples (10 μL) were diluted with purified water (200-fold dilution) and filtered with 0.22-μm filters. On the other hand, the samples for hydrolysis studies were evaporated to dryness under reduced pressure. The dried residues were treated with 100 μL of 6N HCl. After vacuum sealing, the samples were hydrolyzed for 22 hr at 110 °C. After the hydrolysis, the hydrolyzed residues were evaporated to dryness under reduced pressure. The dried residues were dissolved with 200 μL of purified water and filtered with 0.22-μm filters. The filtrate samples were diluted with purified water (50-fold dilution). Both the aqueous samples and hydrolyzed samples were analyzed with an aminoacid analyzer, L-8500 (Hitachi High Technologies Co.), based on the absorbencies of 570 nm and 440 nm after ninhydrin reaction.

### 3.3. LC-MS analysis

LC-MS analysis of the 60 °C sample using pyruvic acid was performed using LCMS-IT-TOF (Shimadzu Co.). The LC-MS components included a LC-20AD pump, a CBM-20A system controller, SPD-20A UV detector. The column used for reversed-phase LC analysis was a TSKgel ODS-80Ts (Tosoh Co.) packed with 5 μm particles (2.0 × 250 mm) and it was incubated at 40 °C.

The LC analysis consisted of the following conditions: Mobile phase A: 100% H_2_O (0.1% formic acid); Mobile phase B: 80% ACN + 20% H_2_O (0.1% formic acid); Flow rate: 0.200 mL/min; Gradient: 0 min 1% B, 10 min 1% B, 40 min 65% B, 40.01 min 100% B, 50 min 100% B, 50.01 min 1% B, and 60 min 1% B. Stop time: 60 min. A 100 μL of injection volume was used. The LCMS-IT-TOF was operated under the following conditions: ESI in positive mode; Detection voltage: 1.53 kV; MS scan range: 80 – 800 m/z. Both amino-acid analyses and LC-MS analyses were conducted at Toray Research Center. Inc.

## 4. Conclusions

**Figure 4.**
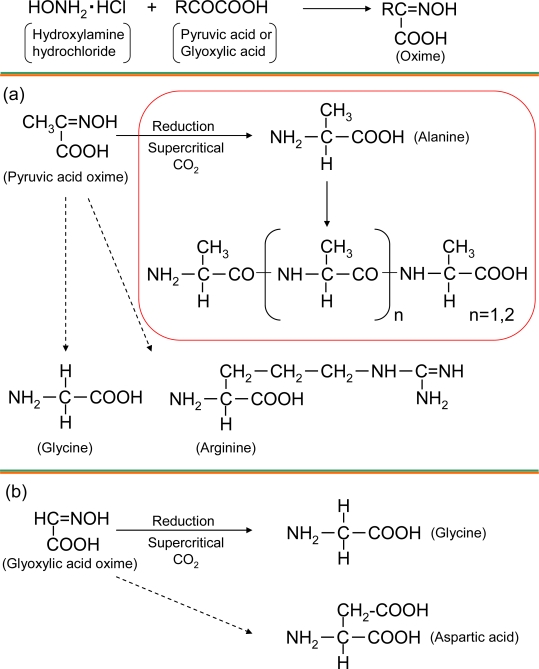
Reaction overview of the supercritical CO_2_/liquid H_2_O reactions. (a) Hydroxylamine + Pyruvic acid (Pyruvic acid oxime) (b) Hydroxylamine + Glyoxylic acid (Glyoxylic acid oxime).

In this research, we have demonstrated that four kinds of amino acid including alanine, glycine, aspartic acid, and arginine were obtained from hydroxylamine hydrochloride and pyruvic acid or glyoxylic acid under supercritical CO_2_ condition, mimicking a soda fountain (Figure 4). Additionally, trimer and tetramer alanine polymers from pyruvic acid were abundantly detected under supercritical CO_2_ conditions, but glycine polymers were almost not detected. From these data, we conclude that even at low temperatures less than 60 °C, supercritical CO_2_ and liquid H_2_O conditions could provide a variety of amino acids and short polymers when the formations are stable. Although the reaction mechanism was not revealed clearly (e.g. which factor is more important, temperature or pressure?), our research opens a new pathway in the study of the origin of life.

## Figures and Tables

**Table 1.** Products obtained from keto acids and hydroxylamine in supercritical conditions (60 ºC) or subcritical condition (31 ºC) before/after hydrolysis reaction.

Primary Reactant	Temp. (ºC)	Products (μmol)							
		Before hydrolysis reaction				After hydrolysis reaction			
		Ala	Gly	Asp	Arg	Ala	Gly	Asp	Arg
Pyruvic acid	60	60.7	0.99	—	—	256.2	1.5	—	0.32
	31	4.1	2.2	—	—	70.2	3.4	—	—
Glyoxylic acid	60	—	866.3	2.5	—	—	819.2	1.8	—
	31	—	183.3	—	—	—	209.2	—	—

— indicates “Not Detected”.

**Table 2.** Polymers obtained from pyruvic acid and hydroxylamine in supercritical conditions.

	Alanine monomer	Dimer	Trimer	Tetramer	Pentamer	Hexamer	Heptamer
Calculated [M+H]^+^	90.05	161.09	232.13	303.16	374.20	445.24	516.28
Retention time(min)	suspended	—	33.7	34.6	—	—	—

— indicates “Not Detected”. The retention time for alanine monomer in the sample could not be specified with the LC-MS system used.
